# False positive results in the Widal test in adults immunized with the Commirnaty (Pfizer-BioNtech) and BBIBP-CorV (Sinopharm) vaccines against COVID-19

**DOI:** 10.3389/fmed.2025.1592019

**Published:** 2025-08-21

**Authors:** Jeel Moya-Salazar, Lizbeth Y. Ciprian, Víctor Rojas-Zumaran, Belén Moya-Salazar, Eliane A. Goicochea-Palomino, Hans Contreras-Pulache

**Affiliations:** ^1^Faculties of Medicine, Universidad Señor de Sipán, Chiclayo, Peru; ^2^Clinical Laboratory Area, Clínica Celestial de Ayacucho, Ayacucho, Peru; ^3^Department of Pathology, Hospital Nacional Docente Madre Niño San Barotlomé, Lima, Peru; ^4^School of Medicine, Universidad Norbert Wiener, Lima, Peru; ^5^Infectious Unit, Nesh Hubbs, Lima, Peru; ^6^Faculties of Health Science, Universidad Tecnológica del Perú, Lima, Peru

**Keywords:** COVID-19, false positive, vaccine, Salmonella, Widal test, SARS-CoV-2, *in vitro*, Peru

## Abstract

**Introduction:**

Vaccination against COVID-19 has generated a dramatic reduction in deaths and infections worldwide. However, there may be cross-reactivity with numerous biochemical and immunological markers. The Widal test for the detection of typhoid fever is an antigen–antibody test that can be affected by vaccination, causing errors in the results, so we determined the frequency of false positive results of the Widal test in adults vaccinated with Commirnaty (Pfizer -BioNtech) and BBIBP-CorV (Sinopharm) vaccines.

**Methods:**

We conducted a cross-sectional study analyzing the titers of the serum agglutinins (*S. typhi* O and H antigen, and *S. paratyphi* A and B antigen) in 50 adults.

**Results:**

The proportion of false positives for O, H, A, and B antigens was 60, 44, 8, and 40%, respectively (total false positives = 38%). The Wildal tests’ results of patients with Commirnaty vaccine had higher false-positive dilution titers for O antigen [titer 1/400 (7.1%) and 1/800 (9.5%)] and H antigen [titer 1/400 (4.8%) and 1/800 (17.6%)]. We found differences in the paratyphic B by age and gender (*p* < 0.05).

**Conclusion:**

Our results suggest misleading results of the Widal test in patients vaccinated against COVID-19, mainly in those vaccinated with Commirnaty. It is important to monitor and evaluate the results of routine immunological tests to ensure the quality of medical care.

## 1 Introduction

Almost a year after the World Health Organization (WHO) declared COVID-19 infection to be a world health emergency, the production of vaccines against SARS-CoV-2 escalated, which allowed the unprecedented start of secondary prevention programs by the end of 2020 (1). Since then, all the countries have developed their immunization plans and have been promoting the COVID-19 vaccination, which have various available commercial types and booster doses (2).

The Pfizer-BioNTech COVID-19 Vaccine (CONMIRNATY) has been one of the most accepted, safe, and available in most of the countries in the midst of 2021, which made it be one of the most used (3). However, in Peru, Sinopharm vaccines (BBIBP-CorV) have been initially distributed among the vulnerable populations such as the armed forces, health personnel, and older people (4). In the midst of corruption scandals and political problems (5, 6), Peruvian vaccination programs have used BBIBP-CorV vaccines, achieving the reduction of severe cases and deaths due to COVID-19, even when there are differences in the composition of each vaccine (7).

With the use vaccines, cross-reactivity has been reported in COVID-19 patients with serological markers, among them, the serological test for the detection of typhoid fever (8, 9). It is possible that, due to the production of antibodies because of the use of vaccines against COVID-19, interferences with other diagnostic tests based on antigen–antibody reaction might have been generated, which could have caused false-positive results. Cross-reactivity in vaccinated patients with more than one dose, which progressively produces a higher level of antibodies against SARS-CoV-2, could also interfere with tests based on antigen–antibody reactions such as the Widal test (typhoid fever) for salmonellosis (10). In Peru, research that quantifies the frequency of false positive results in patients vaccinated against COVID-19 has not been carried out. Hence, the typhoid fever test could produce uncertainty with regard to their results in the context of a mass vaccination of the population. In addition, the Peruvian population has a high risk for typhoid fever (11) and, therefore, understanding the frequency of false positive results, what types of altered serological markers there are, and in what proportion these cross-reactions appear is unavoidable.

We aimed to determine the frequency of false positive results of the Widal test for adults vaccinated with Commirnaty and BBIBP-CorV vaccines against COVID-19. During the pandemic, Peru was among the first countries to deploy BBIBP-CorV vaccines to its population. Despite the clinical trials being intentionally modified, the Peruvian government still approved and used the vaccine, overlooking certain details about its efficacy, side effects, and physiological responses. The hypotheses of this study were the following: (i) there exists a high frequency of false positive results in patients vaccinated with BBIBP-CorV and Commirnaty; (ii) there exist differences between the types of COVID-19 vaccines used; and (iii) patients with post-immunization adverse events have presented with higher titers of typhoid fever. Taking into account the need to achieve a quality assurance of laboratory tests, this study is certainly important in order to achieve the continuous improvement of laboratory processes during the COVID-19 pandemic.

## 2 Materials and methods

### 2.1 Study design, population and inclusion criteria

This cross-sectional study was developed in the Clinical Laboratory Area of the Celestial Clinic, in the Municipality of Ayacucho (2,746 meters above the sea level) in Peru. All the patients attended the occupational health consultation for their monthly work evaluation; hence, none of them presented with fever or symptoms related with salmonellosis in the initial medical check-up. Vital functions (i.e., blood pressure, temperature) were assessed at the clinic triage and and then underwent a comprehensive clinical evaluation in general medicine offices. The inclusion criteria were the following: healthy patients over 18 years of age of both genders, admitted for their routine control of COVID-19, vaccinated against COVID-19 (Commirnaty and BBIBP-CorV) during 2021, and with Widal test’s result for typhoid fever. We excluded foreign patients, those with a recent diagnosis of COVID-19, pregnant females, patients with hypertensive or tuberculosis treatment, with dyslipidemias or diabetes. As this study evaluated all patients treated in 2021, the sample was non-probabilistic and convenience sampling to cover the total number of patients treated.

### 2.2 Vaccination and Widal slide agglutination test

To identify the immunized patients, they were asked to show their vaccination cards in physical format and the doses were verified in the vaccination system of the Ministry of Health.^1^ The immunized patients were included in the study by filling out the written informed consent. The Widal test was administered following the immunoserology recommendations guide of the Ministry of Health (12), and the data were included in a data collection card designed by the authors.

### 2.3 Variables, data gathering, and analysis

Demographic variables were considered (age, gender, residence, occupation), variables of immunization (vaccine type, dose, vaccination date, adverse events, and use of post-vaccination analgesics), and results of the Widal test (for example, titer of serum agglutinins -antibodies against Salmonella antigens: O-somatic and flagellar H-). An early-morning sample of blood (3–4 mL) was taken from the patients following the guidelines of CLS H18-A4 between September and November, 2021 (13).

For agglutination on slides of the Widal test, we used QCA reagents (Barcelona, Spain). We used two drops of centrifuged blood serum with one drop of O and H antigens and parathypic A and B. Then it was shaken for 2 mins in an orbital shaker, and the formation of the antigen–antibody reaction was determined by visible agglutination on the card (positive result). We considered for each positive sample, serial dilutions of the serum of 1:20, 1:40, 1:80, 1:160, and on. The positive titer value is determined by the formation of visible clumps at 1:80 or higher dilutions from the mixture of each antigen with the patient’s serum. To ensure the quality of the results, positive and negative controls were performed using commercially available reagents (14). All results were confirmed using another antibody test from a different manufacturer (IgG/IgM Montest Rapid Test, Mont Group, Lima, Peru). The method used to identify false positive results was based on the difference between the antibody estimate and the Widal agglutination test.

We used the IBM SPSS v24.0 (Armonk, United States) for data analysis. Initially, we used descriptive statistics for the estimation of averages and standard deviation for the continuous variables and measures of frequency and central tendency for categorical variables. The titers of the serum agglutinins (*S. typhi* O and H antigen, and *S. para*-typhi A and B antigen) were determined following the recommendations of the manufacturer. The Kolgomorov-Smirnov test was used to determine data normality; similarly, the unpaired T test was used to see differences among the false positive results between the vaccines; and, finally, Pearson correlation coefficient test was used to determine the correlation between the characteristics of the administered vaccines and cross-reactivity with serum agglutinins of the Widal test. We used binary logistic regression to determine the predictor variables for these false-positive results and used diagnostic tests to find the specificity, negative predictive value and proportion of false positives for each Widal agglutinins. A threshold of significance of *p* < 0.05 and a confidence interval at 95% were considered for all the tests.

### 2.4 Ethical aspects

Written informed consent has been distributed and administered to study participants. The study has followed the guidelines of the declaration of Helsinki (15) and maintains the safeguarding of the results in accordance with Law 25,717 (Peru’s Data Protection Law) (16). In addition, this study has been approved by the Board of Directors of the Clinic (Oficio N° 101–12–2021-21) and by the Ethics Committee of the Universidad Norbert Wiener (VRI-N-089-2022).

## 3 Results

### 3.1 Demographic data

The average age of the patients was 41.88 ± 9.29 (95% CI: 39.30–44.46) and 46 (92%) were male. A total of 42 (84%) of the patients were vaccinated with the their Commirnaty vaccine as their last dose; 49 (98%) patients received the two doses and the average of weeks between vaccination and the Widal test was 28.54 ± 26.75 days (95% CI: 21.13–35.95). Although 38 (76%) patients did not have post-vaccination adverse events, 45 (90%) had consumed anti-inflammatory drugs (Table 1).

**Table 1:** Baseline characteristics of population enrolled in the study (*N* = 50).

Variable	Categories	N	%	*p*-value
Age group (years)	<30	6	12	0.075
	31–40	14	28	
	41–50	22	44	
	>50	8	16	
Gender	Male	46	92	0.001
	Female	4	8	
Vaccine	Commirnaty	42	84	0.001
	BBIBP-CorV	8	16	
Doses	1	1	2	0.001
	2	49	98	
Adverse event	None	38	76	0.078
	Fever*	3	6	
	Pain**	9	18	
Treartment of symptoms	None	5	10	0.255
	NSAIDs	45	90	

*Includes fever and body ache/headache. **Includes arm, body, and headache. NSAIDs, nonsteroidal anti-inflammatory drugs.

### 3.2 False positive results

**Figure 1:**
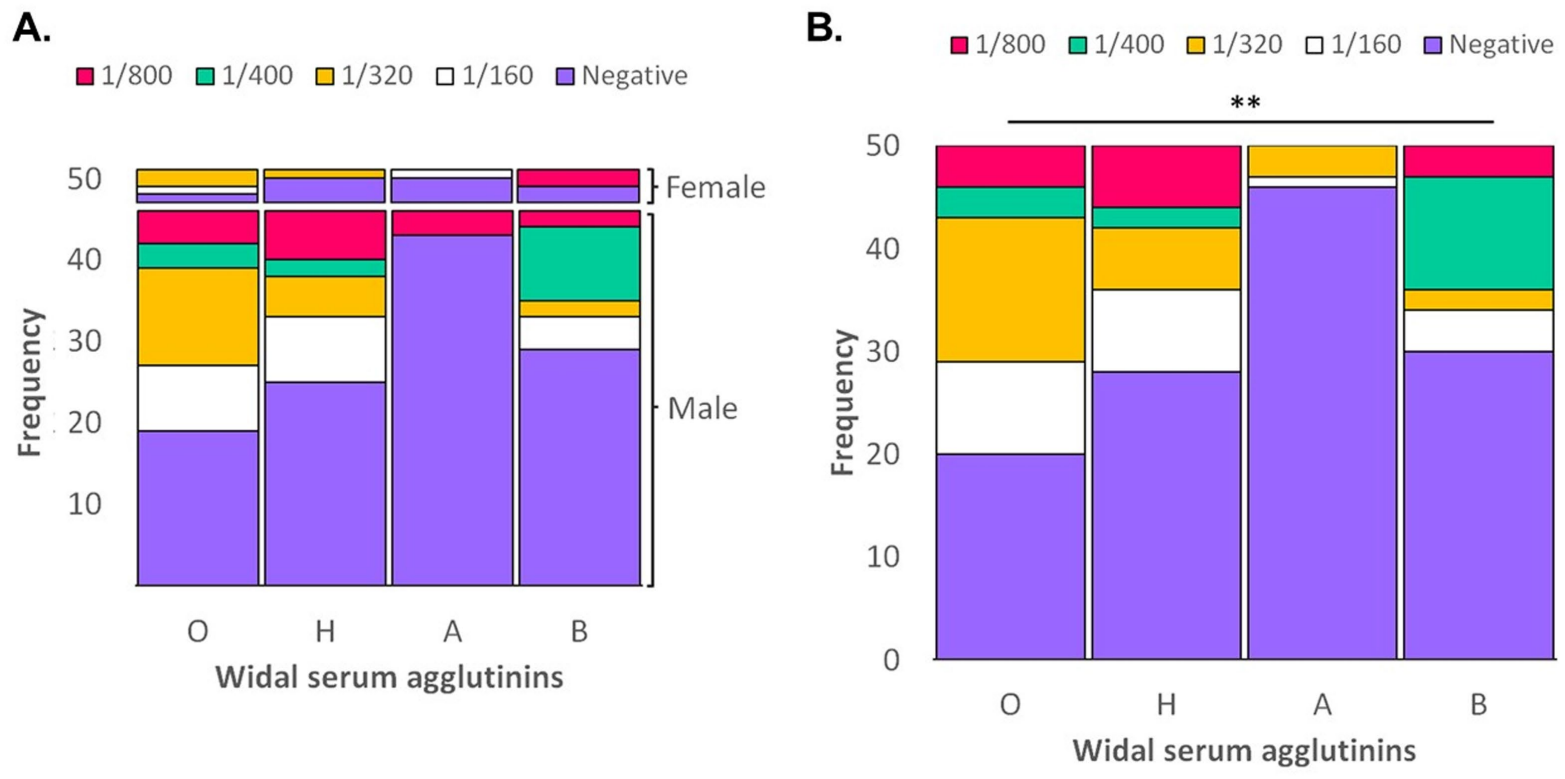
Antibody titer of Salmonella serum agglutinins (Test de Widal) in patients vaccinated against COVID-19. **(A)** Global frequency of dilutions of Salmonella antigens. **(B)** Frequency of dilutions of Salmonella antigens according gender. **p > 0.05.

In total we found 20 (40%) and 28 (56%) patients with negative results for typhoid O and H, respectively, while 46 (92%) patients had negative results for paratyphoid A and 30 (60%) for paratyphoid B. We found no significant differences between Widal’s test results of typhi and paratyphic (*p* > 0.05; Figure 1). The proportion of false positives for O, H, A and B antigens was 60, 44, 8, and 40%, respectively (total false positives = 38%). Likewise, O antigen specificity was 40%; H antigen was 56%; An anti-gen was 92%; and B paratyphic, 60%.

The Wildal tests’ results of patients with the Commirnaty vaccine had higher false positive dilution titers for O antigen [titer 1/400 = 3 (7.1%) and 1/800 4 (9.5%)] compared with BBIBP-CorV vaccine results. For the H antigen, a high frequency of titers was evidenced in 1/400 (two patients, 4.8%) and in 1/800 (six patients, 17.65%) patients who had been vaccinated with the Commirnaty vaccine (Table 2). The BBIBP-CorV vaccine had a low frequency of false-positive results with high titers for paratyphi A (1/800) and B (1/400), both positive in one patient (12.5%). The samples of patients with Commirnaty vaccines showed higher titers against paratyphi B [titer 1/400 = 10 (23.8%) and 1/800 = 3 (7.1%)].

**Table 2:** False positive results and dilution titer of Widal antigen agglutinins according to the type of vaccine administered.

Result/Titer	O-antigen		H-antigen		A-antigen		B-antigen	
	BBIBP-CorV	Commirnaty	BBIBP-CorV	Commirnaty	BBIBP-CorV	Commirnaty	BBIBP-CorV	Commirnaty
Negativo	4 (50)	16 (38.1)	6 (75)	22 (52.4)	7 (87.5)	39 (92.9)	7 (87.5)	23 (54.8)
1/160	1 (12.5)	8 (19)	1 (12.5)	7 (16.7)	0 (0)	1 (2.4)	0 (0)	4 (9.5)
1/320	3 (37.5)	11 (26.2)	1 (12.5)	5 (11.9)	0 (0)	2 (4.8)	0 (0)	2 (4.8)
1/400	0 (0)	3 (7.1)	0 (0)	2 (4.8)	0 (0)	0 (0)	1 (12.5)	10 (23.8)
1/800	0 (0)	4 (9.5)	0 (0)	6 (17.6)	1 (12.5)	0 (0)	0 (0)	3 (7.1
*p*-value	0.418		0.256		0.352		0.804	

### 3.3 Prediction analysis

The analysis of the characteristics of the patients vaccinated against COVID-19 only demonstrated the differences of the paratyphi B with age (*p* = 0.005) and gender (*p* = 0.037). The bivariate analysis showed that the variables included (such as age, vac-cine type, vaccination date) were not predictors of the false-positive results of the Widal test (Table 3).

**Table 3:** Binary regression of the predictive variables of false positive results of the serum agglutinins of the Widal test.

Variables	O-antigen				H-antigen				A-antigen				B-antigen			
	B	SE	*p-*value	95% CI	B	SE	*p*-value	95% CI	B	SE	*p*-value	95% CI	B	SE	*p*-value	95% CI
Age group (years)	1.208	1.029	0.247	−0.865 to 3.281	−0.707	0.933	0.452	−2.585 to 1.171	1.916	1.364	0.167	−0.831 to −4.663	−1.747	0.909	0.061	−3.578 to 0.085
Gender	0.001	0.031	0.974	−0.062 to 0.064	0.012	0.028	0.681	−0.045 to 0.069	−0.011	0.041	0.791	−0.094 to 0.072	−0.055	0.028	0.051	−0.111 to 0.001
Vaccine	−0.016	0.044	0.711	−0.105 to 0.072	−0.071	0.040	0.083	−0.151 to 0.010	0.023	0.058	0.693	−0.094 to 0.140	−0.052	0.039	0.187	−0.130 to 0.026
Dosis of vaccine	0.016	0.016	0.349	−0.018 to 0.049	0.012	0.015	0.443	−0.019 to 0.042	0.012	0.022	0.599	−0.032 to 0.056	0.012	0.015	0.398	−0.017 to 0.042
Adverse events	0.040	0.050	0.425	−0.060 to 0.140	−0.052	0.045	0.257	−0.142 to 0.038	−0.047	0.066	0.476	−0.180 to 0.085	−0.051	0.044	0.254	−0.139 to 0.038
Treartment of symptoms	−0.004	0.036	0.904	−0.077 to 0.068	0.019	0.033	0.554	−0.046 to 0.085	−0.010	0.048	0.832	−0.106 to 0.086	−0.010	0.032	0.832	−0.740 to 0.054
Days post vaccination	4.120	3.033	0.181	−1.989 to 10.228	−2.382	2.748	0.391	−7.917 to 3.154	5.555	4.019	0.174	−2.540 to 13.651	2.595	2.679	0.338	−2.801 to 7.992

*p-value <0.05 (significant).

**Figure 2:**
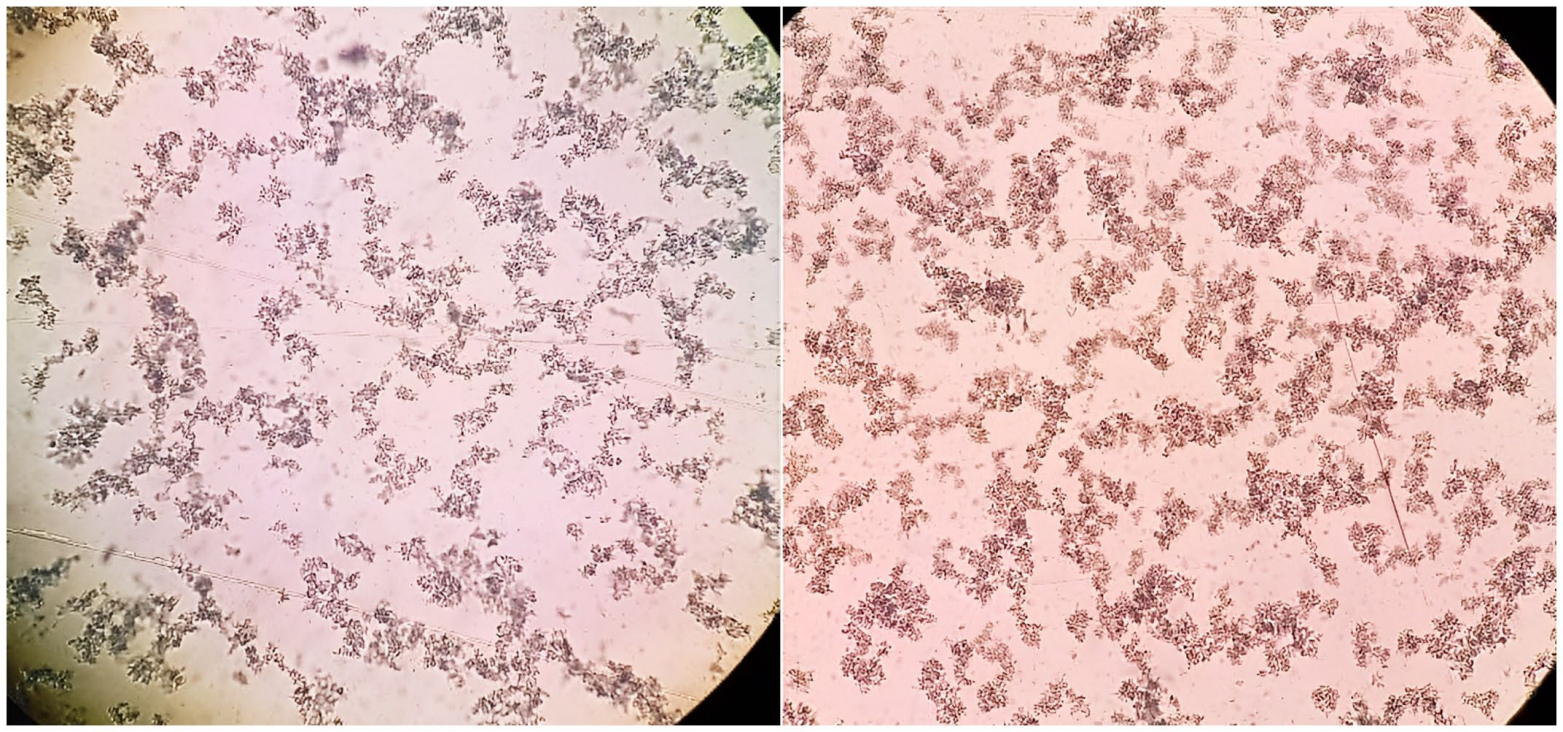
Positive Widal test results in samples from patients vaccinated against COVID-19. In the right the result for Salmonella antigens O-somatic (titer 1/400) is shown, and in the left the positive result for para-typhus A (titer 1/320) is shown.

No patient with a false-positive result for the serum agglutinins was reported, but we did found positivity to the O, H and A antigens and four (8%) patients with positive results for A, H, and B antigens (Figure 2).

## 4 Discussion

In this study, we determined a high frequency of false-positive results of the Widal test in patients vaccinated against COVID-19. O and H typhus presented the most false positive results and with higher dilution titers in comparison with paratyphi (A and B). In addition, although we detected that the highest frequency of false positive results in patients immunized with Commirnaty (Pfizer-BioNtech) vaccines, there were not significant differences between the results of those patients vaccinated with BBIBP-CorV vaccines (Sinopharm). Moreover, the demographic, clinical, and immunization variables were not predictors in regard to false positives.

This study had different strengths. First, to the best of our knowledge, this is the first study that evaluated false-positive seropositivity of the Widal test in patients vaccinated against COVID-19. Although there are numerous studies that have demonstrated that other infectious and chronic diseases can lead to positive results of antigen–antibody tests of serological detection tests of SARS-CoV-2 (8–11, 17) so far, none of them has characterized the effects of immunization in other routine tests such as the Widal test. Second, this study has studied the effects of the Widal test results, by comparing two vaccines extensively used in the vaccination plans against COVID-19. Therefore, the results of the frequency of cross-reactivity of antibody tests with each vaccine can indicate us the subsequent effects and how the laboratories should monitor their processes to ensure reliable results.

The decrease of the rate of infection and deaths due to the vaccines has caused a reduction of the pandemic effects in the world, which allowed the improvement of the restrictions established during 2020–2021 (7). However, as it has been noticed in patients misdiagnosed with a recent SARS-CoV-2 infection (18), there might also exist immune response antibodies which can cause cross-reactivity with other tests based on serology, such as the Widal test or the dengue test (8, 11, 19). This can lead to false-positive results which might affect the quality assurance of laboratory results.

In the beginning of 2020, false-positive dengue results were reported in patients with COVID-19 serology. Yan et al. (9), through their study in Singapore, described a number of cases with serological rapid tests with positive results for dengue’s IgM and IgG antibodies that turned out to be misleading results after administering SARS-CoV-2 tests. In Indonesia, Luhulima et al. (17) showed that a patient aged 43 who suffered from dengue haemorrhagic fever had a positive COVID-19 result after being administered a serological ELISA IgG and IgM test. Subsequent determination indicated the presence of IgG antibody due to dengue which caused the cross-reaction antibody (17). Also, the study by Boukli et al. (8) reported a high incidence (10%) of false-positive results, which led to discontinuation of the use of the commercial chemiluminescent microparticle immunoassay Liaison SARS-CoV-2 S1/S2 IgG at the Saint-Antoine Hospital (Paris, France). These cross-reactivity antibody results were consistently observed in patients suffering from acute infectious diseases, especially from Epstein–Barr virus or hepatitis B virus infection (7). Consistent with these results on rapid tests and automated equipment, more than 35% of our patients obtained false positives after the administration of the Widal test with the Química Clínica Analítica (QCA) reagent for any of the serum agglutinins tested.

It is clear that there continues to be an overlap in the diagnosis of diseases with the same clinical threshold as COVID-19 at the symptoms level. For Salmonellosis, some cases of co-infection have been reported in low- and middle-income countries (10, 20, 21), but false-positive results have also been reported. Babu and Srees showed that six patients in India had false-positive Widal’s test results for typhoid fever when they developed COVID-19 (8). Our results are partially consistent with the study previously mentioned as they identified false-positive seropositivity in the Widal test post-immunization against SARS-CoV-2. However, our false-positive results are independent of the clinical, demographic, and vaccination characteristics of the patients, and are possibly due to the concentration of antibodies caused by the vaccine and to non-specific antibody binding and activation (22, 23).

The frequency of false positives in patients immunized with Commirnaty (Pfizer-BioNtech) or BBIBP-CorV (Sinopharm) vaccines did not show significant differences, although the dilution titers were higher with the former. The results of the Widal test of patients with the Commirnaty vaccine showed higher false positive dilution titers for 3/4 serum agglutinins (O, H and B), which would indicate a greater humoral response with the Commirnaty vaccine (Pfizer-BioNtech) (24), a greater nonspecific binding of these antibodies during the Widal test, or both. Therefore, it is important to define the performance characteristics and false-positive results of typhoid diagnostic tests during the COVID-19 pandemic in order to achieve quality assurance of clinical testing laboratory.

This study had limitations that must be recognized. First, the study was unicentric, with a population from the Peruvian Andes. Therefore, there might be differences with the results in regard to other populations. Second, the Widal test was used in this study; however, the performance and the proportion of false-positive results may vary between commercial kits. Third, we compared cross-reactivity results for the Widal test post immunization with Commirnaty (Pfizer-BioNtech) or BBIBP-CorV (Sinopharm) vaccines; however, there are other available vaccines that could change the false-positive rate and performance. In addition, booster doses of vaccines continue to increase during the pandemic, which could impact the production of antibodies that cause the interference of results of the Widal and other tests.

## 5 Conclusion

In conclusion, this study demonstrated, for the first time, the misleading results of the Widal test in patients vaccinated against COVID-19. The proportion of false-positive results was higher in those vaccinated with Commirnaty (Pfizer-BioNtech) but showed no notable differences and it seems that clinical, demographic, and vaccination characteristics are not predictors of these results. Thus, serological tests for the detection of typhoid fever may be affected, causing false-positive results and compromising the diagnostic processes of health centers, which might generate delays in the detection of this pathogen, the use of other diagnostic tests, and diagnostic uncertainty in patients and physicians.

## Data Availability

The original contributions presented in the study are included in the article/supplementary material, further inquiries can be directed to the corresponding authors.

## Footnotes

^1^ https://carnetvacunacion.minsa.gob.pe/#/auth
